# Willingness to donate genomic and other medical data: results from Germany

**DOI:** 10.1038/s41431-020-0611-2

**Published:** 2020-04-01

**Authors:** Torsten H. Voigt, Verena Holtz, Emilia Niemiec, Heidi C. Howard, Anna Middleton, Barbara Prainsack

**Affiliations:** 1 Institute of Sociology, RWTH Aachen University, Aachen, Germany; 2 Centre for Research Ethics and Bioethics, Uppsala University, Uppsala, Sweden; 3 Society and Ethics Research, Connecting Science, Wellcome Genome Campus, Cambridge, UK; 4 Faculty of Education, University of Cambridge, Cambridge, UK; 5 Department of Political Science, University of Vienna, Vienna, Austria; 6 Department of Global Health & Social Medicine, King’s College London, London, UK

**Keywords:** Ethics, Genetics

## Abstract

This paper reports findings from Germany-based participants in the “Your DNA, Your Say” study, a collaborative effort among researchers in more than 20 countries across the world to explore public attitudes, values and opinions towards willingness to donate genomic and other personal data for use by others. Based on a representative sample of German residents (*n* = 1506) who completed the German-language version of the survey, we found that views of genetic exceptionalism were less prevalent in the German-language arm of the study than in the English-language arm (43% versus 52%). Also, people’s willingness to make their data available for research was lower in the German than in the English-language samples of the study (56% versus 67%). In the German sample, those who were more familiar with genetics, and those holding views of genetic exceptionalism were more likely to be willing to donate data than others. We explain these findings with reference to the important role that the “right of informational self-determination” plays in German public discourse. Rather than being a particularly strict interpretation of privacy in the sense of a right to be left alone, the German understanding of informational self-determination bestows on each citizen the responsibility to carefully consider how their personal data should be used to protect important rights and to serve the public good.

## Introduction

Biomedical research and practice are becoming increasingly data driven. Reasons for this lie both in the demand and in the supply side. On the demand side, the idea that medicine and healthcare should be “personalised” in the sense that prevention, diagnosis, and treatment should be tailored to the individual characteristics of people, means that more data from people are needed. This includes both genomic and other molecular data, biophysical data, and most recently also increasing volumes of behavioural data that is seen to characterise people’s “lifestyle”. On the supply side, ever wider aspects of people’s bodies and lives are “datafied”—that is, they are captured in digital data, via portable or wearable sensors [1]. This means that increasingly large and varied data sets are available that could, in principle, be utilised for medical practice and research.

The mere availability of these data, of course, does not guarantee its quality or utility. Recent scholarship has emphasised the labour and resources needed to make data discoverable, intelligible, and interoperable [2, 3]. Scholars have also warned that “big data won’t cure us” [4]. The fact, however, that these data are available creates pressure to use them to improve individual and/or public health [5].

Besides the urgency to improve the interoperability, interpretation, and utility of this data, this development raises manifold questions as to how to share and govern the use of these data, and for whose benefit. There is a wide agreement among scholars that regulation of genomic and medical data sharing should be informed, in some way, by how people would like their data and information to be used. Producing such empirical evidence on people’s views and preferences is the purpose of the “Your DNA, Your Say” (YDYS) study, a collaborative large-scale survey that is currently available in 14 languages. First results from the English-language survey have been published [6, 7]. Respondents to the English-language arm were based in four countries: the United Kingdom, the United States, Canada, and Australia.

In this article, we report findings from respondents to the German-language version of the survey who resided in Germany at the time of their participation. The German-language branch of the study is led by the first and last author of this paper (THV and BP). Germany is an interesting case in terms of public attitudes towards medical and genetic innovations and the willingness to donate and share medical and genomic data. In the past, it had been shown that the German public tends to be rather sceptical towards genetic testing [8–10]. More recently though, studies found that Germans are open to the broad use of medical data and data sharing [11–13]. In light of these ambivalent findings, it is important to learn more about peoples’ willingness to donate medical and genomic data, to share it with doctors and allow for it to be used for research by non-profit and for-profit organisations.

## Methods

### Survey development

The survey was developed by the Participant Values Task Team (PVTT) from the Global Alliance for Genomics and Health, an international group of researchers (both non-profit and for-profit), policy makers, clinicians, and patient representatives, all with expertise in scientific, legal or social aspects of clinical, and/or research genomics. Survey items were primarily designed by Anna Middleton, Heidi Howard, and Emilia Niemiec, with input from the PVTT (for details see Ref. [14]).

The first and senior author on this paper, who are both fully fluent in English and German, translated the English survey into German. They did this by initially translating the survey from English to German independently and then comparing the translations and discussing all instances where there were discrepancies between the two translations. The German text was subsequently back translated to English by these authors and a number of volunteers to identify further possible ambiguities and inconsistencies, upon which further adjustments were made. About a dozen bilingual colleagues—some of whom were familiar with the YDYS project while others were not—read and commented on various drafts of the translation before it was finalised.

### Sample

Using a market research company, Dynata^1^, we collected completed surveys from a representative sample of individuals in Germany (*n* = 1506). Dynata adheres to the ESOMAR market research code of conduct and is able to recruit participants from nationally representative research panels from across the world. Participants were paid a small financial reward (<€1) for participating. In total, 4391 participants started the survey with 1506 completing it, which is a completion rate of 34.3%. Dynata did not provide information on how many people they invited to take the survey. Where sections of the population were under-represented, Dynata contacted additional respondents to more closely approximate population data. All participants whose data were analysed for this paper filled in the survey between 19 August and 3 September 2017.

### Measures

Our online survey consists of 29 questions, all of which have multiple choice answers and some of these also have an additional request to elaborate in free text. Questions and answers are the same as the English-language version. Data from log files show that it took respondents an average of 35 min to complete the survey. The full survey can be accessed at www.YourDNAYourSay.org. Detailed information on the survey, including the survey design, the short videos that precede different sets of questions and explain basic concepts of genomics and data sharing in non-technical terms, the methodology as well as data storage, protection and privacy and a discussion of some limitations of the survey can be found in a separate article [14, 15].

### Statistical analysis

Sample characteristics were analysed using standard descriptive statistics. Multivariate correlation analyses were used to study effects between different variables, most notably to investigate the association between the willingness to donate DNA for research by different entities and: (1) the personal perception of the status of genetic information (genetic exceptionalism); (2) familiarity with genetics; (3) perspective on harms associated with linking DNA to other personal information. Age, gender, marital status, having children, education level, and religiosity were included as covariates.

We only ran statistical analyses for the German sample, which this paper focuses on. In case of comparison with the English language sample, we used the statistical results published in the respective articles and provide references to those data sets and statistics.

## Results

### Sample description

Regarding age, gender, and education, the distribution in the sample (*n* = 1506) resembles the general population in Germany according to the 2011 census (Table 1). For historical reasons, race or ethnicity is not included in the German census. In our sample, 93% of participants identified as white. Because of the low absolute numbers (*n* = 56) of respondents who identified as non-whites in our sample, we did not use race or ethnicity as a variable for the analysis.

**Table 1:** Sample characteristics by perspective on seeing DNA information as the same/unsure or different to medical information (‘genetic exceptionalist’ views) (*N* indicates count; % indicates percentage).

Variable	Categories	Total (*n* = 1506)		Willing (*n* = 844)		Unwilling (*n* = 257)		Unsure (*n* = 405)		*p*
		*N*	%	*N*	%	*N*	%	*N*	%	
Genetic exceptionalist views	No	865	57.44	438	50.64	164	18.96	263	30.40	<0.0001
	Yes	641	42.56	406	63.34	93	14.51	142	22.15	
Genetic knowledge	Unfamiliar	1066	70.78	533	50.00	195	18.29	338	31.71	<0.0001
	Familiar	439	29.15	310	70.62	62	14.12	67	15.26	
	Missing	1	0.07	1	100	0	0	0	0	
Age	30 and under	379	25.17	250	65.96	48	12.66	81	21.37	<0.0001
	31–40	239	15.87	129	53.97	38	15.90	72	30.13	
	41–50	282	18.73	137	48.58	51	18.09	94	33.33	
	51–60	264	17.53	148	56.06	43	16.29	73	27.65	
	Over 60	342	22.71	180	52.63	77	22.51	85	24.85	
	Missing	0	0	0	0	0	0	0	0	
Gender	Female	764	50.73	417	54.58	126	16.49	221	28.93	0.052
	Male	731	48.54	423	57.87	126	17.24	182	24.90	
	Prefer not to say	11	0.73	4	36.36	5	45.45	2	18.18	
	Missing	0	0	0	0	0	0	0	0	
Children	No	704	46.75	400	56.82	123	17.47	181	25.71	0.090
	Yes	781	51.86	438	56.08	127	16.26	216	27.66	
	Prefer not to say	21	1.39	6	28.57	7	33.33	8	38.10	
	Missing	0	0	0	0	0	0	0	0	
Education	Tertiary	462	30.68	284	61.47	82	17.75	96	20.78	0.002
	Secondary	820	54.45	451	55.00	128	15.61	241	29.39	
	Primary or less	224	14.87	109	48.66	47	20.98	68	30.36	
	Missing	0	0	0	0	0	0	0	0	
Religiosity	Not a religious person	967	64.21	526	54.40	182	18.82	259	26.78	0.045
	A religious person	538	35.72	318	59.11	75	13.94	145	26.95	
	Missing	1	0.07	0	0	0	0	1	100	
Relationship	Married/civil partnership/living together	869	57.71	485	55.81	155	17.84	228	26.35	0.186
	Divorced/single/widowed	636	42.22	359	56.45	102	16.04	175	27.52	
	Missing	1	0.07	0	0	0	0	1	100	

### Genetic exceptionalist views and familiarity with genetics

Less than half of our respondents held views of genetic exceptionalism (~43%). Genetic exceptionalism refers to the idea that genetic material and genetic information are considered as categorically different from other types of information and as such may require additional and/or special governance measures (e.g., specific laws for genetic data). For the purpose of this publication we considered the answer “For me, DNA information is different to other medical information” as indicating a genetic exceptionalism view. In the German language sample, this number is ten percentage points lower than among participants in the English-language survey, where over half (52%) of YDYS survey participants subscribed to genetic exceptionalism. Participants had the option to give free text answers on their reasons as to why they consider genetic information exceptional. The three most common reasons were that “it is unique”, it is “the most personal data we have”, and “it clearly only belongs to yourself”. In terms of sociodemographic variables, there was very little variation between those who did and did not hold genetic exceptionalist views. Respondents who held genetic exceptionalist views were more likely to be under the age of 30 (this age group made up 29% of people with genetic exceptionalist views versus 25% in the overall sample) and more likely to have tertiary education (34% versus 31%).

About a third of our participants stated that they were familiar with DNA, genetics, or genomics. Familiarity with DNA, genetics or genomics refers to more than just knowing about DNA but actual experience: “Yes, I’m familiar through my work, personal interests or family/medical history”. The three most frequently reported sources for familiarity about genetics were personal interest in ancestry and genealogy websites (~33%), personal or family related experiences due to a genetic condition (~11%), and familiarity obtained through professional experience outside of the field of genetics (~10%). In contrast to the topic of genetic exceptionalism, where sociodemographic factors did not correlate with specific views, we found that participants who reported previous familiarity with genetics were more likely to: have a university degree (45.9% versus 30.7% in the overall sample), hold views of genetic exceptionalism (52.7% versus 42.6); have no children (50.7% versus 46.8%); be religious (39.8% versus 35.7%) and be under the age of 30 (37.9% versus 25.2%) (Table 2).

**Table 2:** Sample characteristics by being familiar or unfamiliar with genetics (*N* indicates count; % indicates percentage).

Variable	Category	Familiar		Unfamiliar	
		*N*	%	*N*	%
Genetic exceptionalist views	Yes	232	52.73	409	38.37
	No	208	47.27	657	61.63
Relationship	Married/civil partnership/living together	250	56.82	619	58.12
	Divorced/Single/Widowed	190	43.18	446	41.88
Willingness to donate	Yes	311	70.68	533	50.00
	No	62	14.09	195	18.29
	Not Sure	67	15.23	338	31.71
Gender	Female	217	49.32	547	51.31
	Male	216	49.09	515	48.31
	Prefer not to say	7	1.59	4	0.38
Children	Yes	211	47.95	570	53.47
	No	223	50.68	481	45.12
	Prefer not to say	6	1.36	15	1.41
Education	Tertiary	202	45.91	260	24.39
	Secondary	207	47.05	613	57.50
	Primary or less	31	7.05	193	18.11
Ethnicity	White	398	93.65	1009	97.49
	Non-White	27	6.35	26	2.51
Religiosity	Yes	175	39.77	363	34.05
	No	265	60.23	702	65.85
Age	Under 30	167	37.96	212	19.89
	30–60	197	44.77	588	55.16
	60 and over	76	17.27	266	24.95

### Sensitivity towards different types of personal data

When asked whether all personal data—such as holiday photos, bank information, and medical and DNA information—should have the same level of protection, or whether any data type deserved special protection, 58% of our respondents said that they should all be treated the same. A much smaller proportion (34%) said that these types of information deserve different levels of protection (without yet specifying which types of information deserved more, and which ones less protection), and 8% of participants were not sure. When those who said that different data types deserved different levels of protection were asked to rank these data types according to how much protection they deserved, banking information was ranked highest (76%) with medical and DNA information coming second (both were ranked highest by 67% of respondents). Holiday photos were considered least sensitive (13%).

### Willingness to donate medical and genetic data

A bit more than half (56%) of our respondents were willing to donate medical and DNA information, either to medical doctors or to (non- or for-profit) research. Participants were classified as willing to donate if they said they would agree to make their data available to at least one of the three groups: medical doctors, non-profit researchers, or for-profit researchers. This proportion is lower than in the English speaking countries where more than 67% were willing to donate their medical and DNA information in at least one scenario [6].

When asked whether they would donate their DNA and medical information for use by medical doctors, almost half of our respondents (48%) said they would; about one quarter (25%) said they would not, and 28% said they were unsure (Table 3). The proportions were roughly similar in response to the question of whether they would donate their DNA and medical information for use by non-profit researchers, with slightly fewer (42%) giving a straightforwardly positive answer and slightly more saying no (28%; 29% said they were unsure). Our respondents were considerably less willing to make their DNA and medical information available to for-profit researchers: less than a quarter (23%) were willing to do so, while almost half (48%) were not, and 29% said they were unsure. Those who were unsure whether or not to donate their medical or DNA information made no distinction between the potential user group. Those who were willing to donate clearly distinguished between medical doctors and non-profit research on the one hand, and for-profit research on the other, with a clear preference to donate to the former two.

**Table 3:** Sample characteristics by willingness to donate medical and genomic information to medical doctors, non-profit and for-profit research (*N* indicates count; % indicates percentage).

Variable	Categories	Willingness to donate for use by…																	
		For-profit researchers						Non-profit researchers						Medical doctors					
		Yes (*n* = 341)		No (*n* = 729)		Unsure (*n* = 436)		Yes (*n* = 637)		No (*n* = 429)		Unsure (*n* = 440)		Yes (*n* = 719)		No (*n* = 367)		Unsure (*n* = 420)	
		*N*	%	*N*	%	*N*	%	*N*	%	*N*	%	*N*	%	*N*	%	*N*	%	*N*	%
Genetic exceptionalist views	No	163	18.84	419	48.44	283	32.72	318	36.76	258	29.83	289	33.41	369	42.66	225	26.01	271	31.33
	Yes	178	27.77	310	48.36	153	23.87	319	49.77	171	26.68	151	23.56	350	54.60	142	22.15	149	23.24
	Missing	0	0	0	0	0	0	0	0	0	0	0	0	0	0	0	0	0	0
Genetic knowledge	Unfamiliar	187	17.54	535	50.19	344	32.27	390	36.59	321	30.11	355	33.30	462	43.34	267	25.05	337	31.61
	Familiar	154	35.00	194	44.09	91	20.91	246	56.14	108	24.55	85	19.32	256	58.41	100	22.73	83	18.86
	Missing	0	0	0	0	1	100	1	100	0	0	0	0	1	100	0	0	0	0
Age	30 and under	108	28.50	161	42.48	110	29.02	190	50.13	87	25.59	92	24.27	198	52.24	87	22.96	94	24.80
	31–40	61	25.52	102	42.68	76	31.80	93	38.91	62	25.94	84	35.15	114	47.70	56	23.43	69	28.87
	41–50	57	20.21	137	48.58	88	31.21	102	36.17	87	30.85	93	32.98	116	41.13	70	24.82	96	34.04
	51–60	59	22.35	130	49.24	75	28.41	119	45.08	67	25.38	78	29.55	128	48.48	65	24.62	71	26.89
	Over 60	56	16.37	199	58.19	87	25.44	133	38.89	116	33.92	93	27.19	163	47.66	89	26.02	90	26.32
	Missing	0	0	0	0	0	0	0	0	0	0	0	0	0	0	0	0	0	0
Gender	Female	157	20.55	360	47.12	247	32.33	311	40.71	202	26.44	251	32.85	358	46.86	167	21.86	239	31.28
	Male	184	25.17	359	49.11	188	25.72	323	44.19	220	30.10	188	25.72	359	49.11	195	26.68	177	24.21
	Prefer not to say	0	0	10	90.91	1	9.09	3	27.27	7	63.64	1	9.09	2	18.18	5	45.45	4	36.36
	Missing	0	0	0	0	0	0	0	0	0	0	0	0	0	0	0	0	0	0

We investigated the possible influences on people’s willingness to donate and we found two main factors. First, there was variation between those who held genetic exceptionalist views and those who did not. Second, previous familiarity with genetics had a moderating effect on willingness to donate. Participants with genetic exceptionalist views were overall slightly more likely to donate DNA and medical data (63% versus 56%). This difference was observed for all three domains with 55% versus 48% for medical doctors, 50% versus 42% for non-profit, and 28% versus 23% for for-profit research. In line with the overall sample, willingness to donate was the lowest for for-profit research. In contrast, participants who did not hold genetic exceptionalist views were significantly less likely to be willing to donate their medical information, with only 19% willing to donate their DNA and medical information for for-profit research (Table 3).

Similar effects could be observed for participants who considered themselves familiar with genetics. Overall, they were significantly more willing than others to donate DNA and medical information (71%). Their willingness to donate to medical doctors and for non-profit was also very high at 58% and 56%, respectively. Most notably, 35% of participants with previous genetic familiarity were also willing to donate their information to for-profit research. Thus, previous genetic familiarity had an even stronger effect than genetic exceptionalist views on willingness to donate medical information.

### Multivariate analysis of attitudes towards donation

In our multivariable analysis, we used a multinomial logistic regression model to analyse the determinants on willingness to donate. We included the following variables as covariates with possible influencing effects: genetic exceptionalist views, familiarity with genetics, age, gender, having children, education level, and religiosity (Table 4).

**Table 4:** Multivariate analysis of attitudes towards donation.

Variable	Category	Unwilling				Unsure			
		OR	LCI	UCI	*p*	OR	LCI	UCI	*p*
Genetic exceptionalist views	No	Ref.				Ref.			
	Yes	0.71	0.52	0.96	0.026	0.71	0.55	0.91	0.008
Genetic knowledge	Unfamiliar	Ref.				Ref.			
	Familiar	0.634	0.45	0.89	0.010	0.41	0.29	0.56	0.000
Age	30 years and under	0.61	0.40	0.92	0.020	0.66	0.47	0.94	0.020
	30–60 years	Ref.				Ref.			
	60 years and older	1.38	0.96	1.97	0.075	0.79	0.57	1.09	0.152
Gender	Female	Ref.				Ref.			
	Male	0.94	0.70	1.26	0.682	0.81	0.63	1.04	0.098
Children	No	Ref.				Ref.			
	Yes	0.71	0.51	0.99	0.043	0.95	0.71	1.25	0.702
Tertiary education	No	Ref.				Ref.			
	Yes	1.01	0.73	1.40	0.93	0.77	0.57	1.03	0.084
Religious	No	Ref.				Ref.			
	Yes	0.73	0.53	1.00	0.051	1.02	0.78	1.32	0.894

Participants who were unwilling or unsure about donating their medical and DNA information were less likely to express genetic exceptionalist views (odds ratio (OR) 0.71; 95% confidence interval (CI) 0.52–0.96; *p* = 0.026 for unwilling and OR 0.71; CI 0.55–0.91; *p* = 0.008 for unsure). In addition, those who were unwilling were less likely to be familiar with genetics (OR 0.63; CI 0.45–0.89; *p* = 0.01). Participants who were unsure about donating their information were also less likely to be familiar with genetics (OR 0.41; CI 0.29–0.56; *p* < 0.001). We found substantive differences regarding the willingness to donate across reported age groups. Those who were unwilling or unsure about donating had lower odds of being 30 years or younger (OR 0.61; CI 0.40–0.92; *p* = 0.020 for unwilling and OR 0.66; CI 0.47–0.94; *p* = 0.020 for unsure) than those who were willing to do so. Furthermore, we found some sociodemographic differences between those who were willing to donate and those who were unwilling. Participants who were unwilling to donate their medical and DNA information showed lower odds in terms of having children (OR 0.71; CI 0.51–0.99; *p* = 0.043) than those who were willing to do so. Compared with those who were willing to donate, those who were unwilling had also lower odds of being religious (OR 0.73; CI 0.53–1.00; *p* = 0.051). For those who were unsure about donating we found no significant associations with any sociodemographic data.

In summary, those who were unwilling or unsure about donating their information were significantly less likely to view DNA data and medical data as being different to have gathered genetic familiarity so far and be under the age of 30 than those who were willing to donate. In addition, those unwilling to donate seem to be less likely to have children and to be religious than those who were willing to donate.

## Discussion

Three findings from the German sample are particularly striking and merit further discussion: first, that German participants were on average less willing to make their data available for research than participants in the English-language survey; second, that within the German sample, those who were more familiar with genetics, and those who held genetic exceptionalist views, were more willing to donate their data; and third, that those who held genetic exceptionalist views tended to be more educated and younger than the rest of the sample. We will now consider each of these groups in turn.

### Lower willingness to donate in the German than in the English-language sample

As noted, 56% of the respondents in the German sample were willing to donate medical and DNA information to medical doctors, or to (non- or for-profit) research, compared with over 67% of respondents to the English-language survey [6]. The survey data do not give us any insights into the reasons for the lower levels of willingness to make their DNA and medical information available for research. We hypothesise that lower willingness to share data with institutions may be related to the particular prominence of data protection and privacy in public discourses in Germany.

In a 2010 Eurobarometer survey, which included questions about biobanks and data privacy, German respondents articulated the highest level of concern about the protection of their genetic data of all EU countries [10]. The question was included in the Eurobarometer special Report [16]. Since then, one more Eurobarometer, published in 2015, addressed a similar topic, namely data protection [17]. In that survey, German respondents held very sceptical views. Because the survey did not include questions on genetic information specifically, it is not possible to compare attitudes over time.

The high level of concern with regards to sharing medical and genomic information, as well as data privacy may relate to the specific understanding of informational self-determination in Germany, which is not only considered a fundamental right of citizens, but it entails the idea that the strict protection of personal data “is essential for a free and self-determined development of the individual […]. If citizens cannot oversee and control […] what kind of information about them is openly accessible in their social environment, and if they cannot even appraise the knowledge of possible communication partners, they may be inhibited in making use of their freedom” [18]. Also, the German Genetic Diagnosis Act (Gendiagnostikgesetz, GenDG), which entered into force on 1 February 2010, shows a strong focus on the right to informational self-determination. The right to “informational self-determination” is grounded in Art. 2 (1) and Art. 1 (1) of the German Constitution (Grundgesetz), which protect the personal sphere of life, guaranteeing the respect of human dignity, and the right of free development of one’s personality. The concept of informational self-determination was introduced by the German Federal Constitutional Court (Bundesverfassungsgericht) in a ruling relating to personal information. In its decision on 15th December 1983 it held that “[…] in the context of modern data processing, the protection of the individual against unlimited collection, storage, use and disclosure of his/her personal data is encompassed by the general personal rights of the Basic Constitutional Law. This basic right warrants in this respect the capacity of the individual to determine in principle the disclosure and use of his/her personal data. Limitations to this informational self-determination are allowed only in case of overriding public interest”. The Court held that every citizen has a right to control the flux of all information relating to him or her. It also reasoned that the present and future conditions of data processing and collection permit a wide variety of possible abuses against which the individual has to be protected [19].

Interpreted in this light, the greater hesitance of German respondents to make their information available for use by institutions is not necessarily a sign of distrust in institutions, or a lack of willingness to help others. It could also be an expression of people’s perceived duty and role as citizens. The latter may also help to explain why there was a clear preference for making their DNA and medical information available to non-profit research in the German sample (see Table 3), which could be seen as a commitment to using one’s personal data in such a way that it creates public benefits. This interpretation is in line with findings from a qualitative study on NHS England’s care.data programme and the reasons as to why people opt out [20].

### Participants holding genetic exceptionalist views, and those who are more familiar with genetics, are more likely to be willing to make their information available

We also found that participants who held genetic exceptionalist views (63%), and those familiar with genetics (71%), were more likely to be willing to make their DNA or medical information available for use by institutions than the average of the German sample (56%). This is largely in line with findings from the English-language survey, where a very similar, higher proportion of those holding genetic exceptionalist views, and those being familiar with genetics, were more willing to donate their data, with the greatest willingness among those who both held genetic exceptionalist views and were familiar with genetics [7]. In the German sample, previous genetic familiarity had an even stronger effect on people’s willingness to donate than genetic exceptionalist views.

Also here, we have no indication from the survey data as to why those who believe that genetic information is special, and those who consider themselves familiar with this topic, are more willing to make their information available than others. Drawing upon the work of colleagues exploring this issue in other studies, we think that the analysis of Hobbs et al. is particularly relevant [9, 10]. In their work with focus groups in Germany and the United Kingdom, which included biobank participants and members of “general” publics, these authors found that both in the German and British sample, the main reason to participate was the idea that people’s data “contributed to the ‘common good’, typically presented by participants as the progress of medical science or improved population health” [10]. We hypothesise that also in our sample, the more people felt they understood the potential of genetics, and the more they felt that genetic information was “special” in its role to explain health and disease, the more strongly they felt a duty to contribute to health research with public benefits.

Familiarity with DNA, genetics and genomics also increases the willingness to donate medical and genomic information. This finding is in line with other studies such as Balck et al. [9]. While they find that Germans are overall more critical of genetic testing than their comparative sample of Finnish respondents, they also find that education is positively correlated with a more positive attitude towards genetic testing. We think that this is due to the fact that knowing about details of genetics and genomics helps to realistically assess opportunities and risks and increases the willingness to donate personal data.

### Genetic exceptionalists were younger and more educated

The third finding that needs further discussion is that in our sample, respondents who held genetic exceptionalist views were slightly overrepresented under the age of 30 and more likely to have tertiary education (34% versus 31%). The latter finding—that tertiary education corresponded positively with the holding of genetic exceptionalist views—was also present in the larger, English-language sample [7]. A possible explanation for this is that the longer people have been exposed to science education or training, the more they are aware of distinctive features of genetic information, e.g., it is familial and unchangeable. Through this awareness they believe that genetic information is a “special” kind of information, which is different to other personal data. Assuming that many respondents would have been exposed to science education and training during or after the Human Genome Project, where public and also expert discourses focused heavily on the special role and value of genetic and genomic data, this may have contributed to a greater prevalence of genetic exceptionalist views among those who were in education and training for longer.

A correlation that we found in the German sample that was not found in the English-language survey was the association between younger age and genetic exceptionalism. A possible explanation for the correlation of genetic exceptionalism and younger age among Germans could be the current public debate in Germany about the use of genetic information by the police, which is a debate that young people in particular participate in [21]. This could account, in part, for the greater prevalence of genetic exceptionalism in younger people in Germany. It would be necessary to conduct further quantitative and qualitative research in order to substantiate this claim and learn more about the reasons for this correlation.

## Conclusion

This paper reports from the German arm of the YDYS study, a research collaboration to explore understandings and views of publics in many countries and world regions towards the use of their medical, DNA, and other data. On the basis of a representative sample of the German population, our main findings include that the proportion of those holding exceptionalist views is lower (43%) than in the English language part of the survey (52%), and that those holding exceptionalism views are more likely to be young and educated. Also, the proportion of those who are willing to donate their information to research was found to be considerably lower in the German sample (56%) than in the English-language arm (67%), with the willingness to donate to for-profit research being lowest. We also found that those willing to donate (regardless of the purpose of the research) tended to be more familiar with genetics, and more likely to hold views of genetic exceptionalism.

We believe that these findings can best be explained with reference to the special status that data protection has in German public discourse. As noted, the right to information self-determination assumes an important role in German law and for the identity of many Germans. It is not to be understood merely as a strong emphasis on privacy in the sense of a right to be let alone [18]. Instead, it imposes a positive responsibility on people to think carefully about how their data should be used, as exercising control over data is part of one’s autonomy and ultimately also an expression of citizenship. The expression of autonomy through exercising one’s right to informational self-determination is not, in turn, a solipsistic activity where people seek to maximise their own individual gain; on the contrary, they are expected to utilise their data in such a way that it protects their own rights and creates benefits for others or even for the entire collective.

Against the backdrop of the important role that informational self-determination plays in German public discourse it does make sense that the proportion of people holding genetic exceptionalist views are relatively low—because there is great awareness of the need to carefully control the use of all data types, not only genetic data. The same perceived responsibility to carefully think about who one makes her personal data available to could explain the relatively low willingness to donate data for research in the German sample. Those with greater familiarity with genetics, however, and those who believe that genetic data are “special”, are more aware of the benefits that medical research can obtain with the help of genetic data specifically and thus are more willing to donate it.

## Footnotes

1 At the time when the survey was conducted the same company operated under the brand name ResearchNow (https://www.dynata.com)
